# Adherence to Dietary Approaches to Stop Hypertension (DASH) Diet as a Protective Factor for Ischemic Stroke and Its Influence on Disability Level: A Case–Control Study in Lebanon

**DOI:** 10.3390/nu16183179

**Published:** 2024-09-20

**Authors:** Jad El Masri, Hani Finge, Tarek Baroud, Najla Ajaj, Mariam Houmani, Maya Ghazi, Mahmoud Younes, Pascale Salameh, Hassan Hosseini

**Affiliations:** 1 INSERM U955-E01, Institut Mondor de Recherche Biomédicale, Université Paris-Est Créteil, 94000 Créteil, France; consultation.hosseini@gmail.com; 2 École Doctorale Sciences de la Vie et de la Santé, Université Paris-Est Créteil, 94010 Créteil, France; 3 Faculty of Medical Sciences, Lebanese University, Beirut 1533, Lebanon; tarekbaroud01@hotmail.com (T.B.); najla1ajaj@gmail.com (N.A.); mayanghazi99@gmail.com (M.G.); pascalesalameh1@hotmail.com (P.S.); 4 INSPECT-LB (Institut National de Sant e Publique, d’Épidemiologie Clinique et de Toxicologie-Liban), Beirut 1103, Lebanon; 5 Department of Neurology, Faculty of Medical Sciences, Lebanese University, Beirut 1533, Lebanonmyouness55@yahoo.com (M.Y.); 6 Department of Nutrition, Sahel General Hospital, Beirut P.O. Box 99/25, Lebanon; mariamhoumani193@gmail.com; 7 School of Medicine, Lebanese American University, Byblos 1102, Lebanon; 8 Faculty of Pharmacy, Lebanese University, Beirut 1533, Lebanon; 9 Department of Primary Care and Population Health, University of Nicosia Medical School, 2417 Nicosia, Cyprus; 10 Department of Neurology, Henri Mondor Hospital, AP-HP, 94000 Créteil, France

**Keywords:** ischemic stroke, risk factors, DASH diet, hypertension, disability

## Abstract

Background: Hypertension is a major risk factor for ischemic stroke. An important strategy in controlling hypertension is dietary modification. The present study evaluates the effect of Dietary Approaches to Stop Hypertension (DASH) diet on the risk of ischemic stroke. Methods: A case–control study was carried out, including 214 ischemic stroke cases recruited within the first 48 h of diagnosis and 214 controls, divided equally into hospitalized and non-hospitalized participants. Controls were matched to cases based on age and gender. Socio-demographic characteristics were assessed, in addition to adherence to the DASH diet, which was measured using a preconstructed DASH diet index (ranging from 0 (lowest) to 11 (highest)). For stroke patients, Modified Rankin Score (mRS) was measured to assess disability. Results: Smoking, hypertension, hyperlipidemia, atrial fibrillation, and myocardial infarction were significantly associated with ischemic stroke (*p* < 0.001). Higher adherence to the DASH diet was correlated to lower rates of stroke, where cases scored 5.042 ± 1.486 compared to 6.654 ± 1.471 for controls (*p* < 0.001). Eating more grains, vegetables, fruits, dairy products, nuts, seeds, and beans, and lower levels of fat, fewer sweets, and less sodium were associated with lower rates of ischemic stroke (*p* = 0.038 for sweets and *p* < 0.001 for all the remaining), while meat, poultry, and fish did not have any significant effect (*p* = 0.46). A multivariate analysis showed that lower adherence to the DASH diet (*p* < 0.001, OR: 0.526, CI95% 0.428–0.645) was associated with a higher incidence of ischemic stroke and an increased likelihood of having high disability levels (mRS 5–6) (*p* = 0.041, OR: 2.49 × 10^−8^, CI95% 0–2.49 × 10^−8^). Conclusions: The relation between the DASH diet and risk of stroke highlights the necessity for strict adherence to dietary restrictions, suggesting a protective role for the DASH diet in stroke pathogenesis and prognosis.

## 1. Background

Stroke is one of the main causes of death and functional inability worldwide [1]. In the last decade, the global incidence of stroke increased by 20% in low- and middle-income countries such as Lebanon [2]. Stroke has a great impact on the quality of life of patients and their family carers who provide long-term day-to-day care. Not only the patients but also their caregivers need professional attention and support in order to maintain their own physical and emotional health and well-being [3].

Risk factors for stroke can be categorized as modifiable and nonmodifiable. Age, sex, and race are nonmodifiable, while hypertension, smoking, diet, and physical inactivity are the main reported modifiable risk factors, comprising around 90% of all the stroke-related risk factors [4].

Hypertension is one of the major risk factors causing stroke. It can lead to stroke through several mechanisms, such as causing alterations in and damage to the endothelium, which can lead to local thrombi formation and ischemic lesions. Moreover, degenerative changes in smooth muscle cells and endothelium caused by hypertension predispose for intracerebral hemorrhages. Furthermore, hypertension accelerates the arteriosclerotic process, which increases the risk of cerebral lesions related to stenosis and embolism [5].

An unhealthy diet is one of the most important behavioral risk factors for hypertension [6]. The Dietary Approaches to Stop Hypertension (DASH) diet originated early in the 1990s [7]. It underwent several innovative features, where DASH trials were assessed in the field of nutrition and blood pressure research. By shifting an individual’s diet to follow a certain pattern, the DASH diet achieved impressive results in lowering blood pressure [8]. The DASH diet has been advocated as the first-line pharmacologic therapy, along with lifestyle modifications [7]. This type of diet is easier to follow compared to other types, as it targets major food groups such as vegetables, fruits, carbohydrates, low-fat dairy products, lean meat products, nuts, and grains. Another important point is that the DASH diet promotes a high intake of potassium (K), calcium (Ca), magnesium (Mg), and fibers [9].

In addition to treating hypertension, the DASH diet helps lower blood glucose levels, triglycerides, low-density lipoprotein (LDL-C), and insulin resistance, making it a management strategy in metabolic syndromes. Through these effects, this diet could potentially aid in preventing atherosclerosis, leading to a decrease in the incidence of consequent diseases, such as ischemic stroke [10].

There is evidence that shows that the DASH diet lowers the risk of stroke directly and through acting on risk factors, yet there are limited studies on its effectiveness [11]. Therefore, this study aims to assess the relation between the DASH diet and ischemic stroke, in addition to its effect in the presence of other risk factors. Also, this study aims to assess the effect of DASH diet adherence on disability levels in ischemic stroke patients, confirming its predicted role in decreasing ischemic stroke.

## 2. Materials and Methods

### 2.1. Study Design

A case–control study was carried out to assess the effect of DASH diet adherence on ischemic stroke. An informed consent form was given to all participants, outlining the study’s objectives, benefits, and concerns, and assuring the confidentiality of the collected information. Participation was entirely optional. Information was gathered between February 2023 and December 2023 by collecting clinical information from patients’ medical records and a face-to-face interview to assess dietary habits.

### 2.2. Participants

All included cases were Lebanese people, aged 18 and above, admitted due to an incidence of ischemic stroke to Sahel General Hospitals or Al Rassoul Al Azam Hospital in Beirut.

Inclusion criteria: To be included, patients must have been recruited during the first 3 days following stroke, while the patient is still in the observation period. The diagnosis must be confirmed by computed tomography (CT) and/or magnetic resonance imaging (MRI). Clinical confirmation of the diagnosis was also needed before including each case [12]. Controls were gender- and age-matched, with no clinical indications of stroke, no history of stroke, nor any known risk factors correlated to hypercoagulability or other states linked to cerebrovascular diseases. Controls were recruited from the same hospitals (48%), including patients attending outpatient clinics for illnesses not related to cerebrovascular diseases such as acute kidney injury, bone fractures, COPD exacerbation, pneumonia, urinary tract infection, cataracts, and diabetic ketoacidosis, or from the general population (52%), including visitors or relatives of patients.

Exclusion criteria: The exclusion of cases was based on the absence of consent, the absence of clinical confirmation or a CT/MRI, and the presence of other types of cerebrovascular attack (CVA), such as transient ischemic attack or hemorrhagic stroke [13]. For controls, exclusion was based on the absence of consent and admission for a cause that is a directly related risk of stroke.

### 2.3. Variables and Data Source Measures

The questionnaire was filled out using patient’s medical file records and via a face-to-face interview that took around 20 min to answer.

The socio-demographic characteristics of each participant were assessed, such as age, gender, marital status, education, and employment, in addition to pre-existing health-related conditions, such as smoking, family history of stroke, hypertension (systolic blood pressure > 130 mm Hg or diastolic blood pressure > 80 mm Hg [14]), hyperlipidemia (elevated lipid levels: cholesterol > 200 mg/dL or triglyceride > 15 mg/dL [15]), deep vein thrombosis (DVT) or pulmonary embolism (PE) (blood clots venous circulation or pulmonary circulation [16]), atrial fibrillation (cardiac electricity disturbance [17]), migraine (type of headache [18]), and myocardial infarction (MI) (decreased blood flow to the myocardium [19]).

For ischemic stroke patients, the level of disability was measured using the Modified Rankin Scale, which classifies each patient based on their disability level, ranging from 0 (no disability) to 6 (death) [20].

To determine DASH diet adherence, a Food Frequency Questionnaire (FFQ), which targets 168 foods, was classified into the 11 different food groups (total grains, whole grain, vegetables, fruits, dairy food, meat—poultry and fish, nuts—seeds and dry beans, %kcal from fat, %kcal from saturated fatty acids, sweets, and sodium [21]. Each of the 11 groups was divided into three levels, where the best adherence was denoted by 1 point, moderate adherence by 0.5 points, and worse adherence by 0 points. The total score ranges from 0 (least adherent) to 33 (most adherent). This method is used and accurately explained in another study [21]. Furthermore, the DASH score was categorized into either three (low, moderate, and high adherence) or four categories (low, low–moderate, high–moderate, and high adherence) by dividing the score into equal categories.

### 2.4. Ethical Considerations

This study respects confidentiality and anonymity. Ethical approval was granted from the Institutional Review Board (IRB) at the hospitals included in the data collection (ID number: 1/2023).

### 2.5. Statistical Analysis

Data were analyzed using SPSS software version 25. A descriptive analysis was performed using frequencies and percentages for categorical variables and means and standard deviations for continuous variables. A bivariate analysis was carried out to identify potential risk factors for ischemic stroke, including the adherence to a DASH diet. Student’s test was utilized to compare means between two groups, and the ANOVA test to compare means between more than two groups. The Chi-square and Fisher exact tests were used to compare percentages between two groups. Simple linear regression was used to correlate between continuous variables. A *p* < 0.05 was considered statistically significant.

A binomial logistic regression model was performed to investigate the odds ratio (OR) with a 95% confidence interval (CI) for marital status, education, smoking, family history, hypertension, hyperlipidemia, atrial fibrillation, myocardial infarction, and DASH diet adherence level among participants with ischemic stroke and the control group. The Hosmer–Lemeshow test was non-significant, demonstrating the test’s adequacy. All covariates with a *p* < 0.2 in the bivariate analysis were included in the logistic regression model. The CI was set at 95%, and a value of *p* < 0.05 was considered significant.

Additionally, a multinomial logistic regression was conducted, taking the levels of disability, as measured by the mRS scoring system, as the dependent variable. All variables with a *p* < 0.2 were included in the final model as independent variables, including marital status, education, employment, age, smoking, hypertension, hyperlipidemia, DVT or PE, atrial fibrillation, migraine, myocardial infarct, and DASH diet adherence level.

## 3. Results

### 3.1. Effect of Socio-Demographic Factors on Ischemic Stroke

Table 1 shows the socio-demographic characteristics of cases and controls in this study, where a total of 428 participants, divided into 214 cases and 214 controls, were included. The mean age of cases was 68.589 ± 13.436, and that of controls was 66.841 ± 14.488; *p* = 0.196. Males were predominant in both groups, having a percentage of 51.86 in the cases and 50.47 in controls (*p* = 0.772). There was a significant difference in marital status and education between cases and controls (*p* value < 0.001), while no significance was reported in employment (*p* value = 0.65).

### 3.2. Effect of Socio-Demographic Factors on DASH Diet Adherence

Table 2 shows the level of adherence to the DASH diet in different socio-demographic groups. Females had a significantly higher level of adherence (6.16 ± 1.611) compared to males (5.55 ± 1.7); *p* < 0.001. Having a non-healthcare-related education and being employed were associated with significantly lower levels of adherence (*p* = 0.044 and *p* = 0.026, respectively). There was no significance reported in marital status and age (*p* value = 0.976 and 0.084, respectively).

### 3.3. Effect of Pre-Existing Health-Related Conditions Associated with Ischemic Stroke

Table 3 shows the relationship between ischemic stroke and several factors. Smoking was significantly associated with a higher risk of ischemic stroke, where 126 (58.88%) of cases were smokers compared to 88 (41.12%) of the controls (*p* < 0.001). Additionally, 105 (49.06%) of cases had a positive family history of stroke, versus only 29 (13.55%) controls (*p* < 0.001). Regarding hypertension, more than 90% (194 participants) of the cases were hypertensive while only half of the controls were hypertensive (*p* < 0.001). When compared to controls, cases had a higher rate of hyperlipidemia compared to controls (151 (70.56%) vs. 81 (37.85%); (*p* < 0.001)). Patients with atrial fibrillation also had a higher risk of having an ischemic stroke (34.58%) than the controls (6.07%) (*p* < 0.001). Similarly, the risk of developing stroke in patients with MI was higher in cases (49 cases, 22.90%) compared to controls (17 controls, 7.94%) (*p* < 0.001). Having DVT/PE or migraine was shown to have no significant difference between cases and controls (*p* = 0.111 and *p* = 0.21, respectively).

### 3.4. Effect of DASH Diet Adherence on Ischemic Stroke

**Figure 1:**
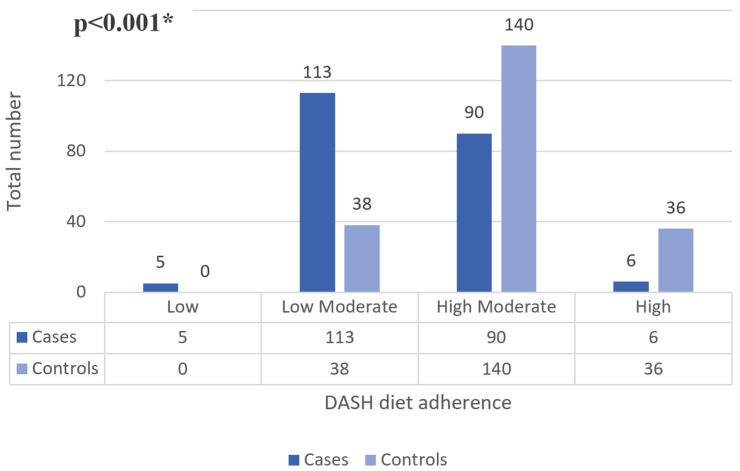
Descriptive and bivariate analysis of DASH diet adherence level on ischemic stroke. * represents *p* < 0.05.

Figure 1 shows the level of adherence to the DASH diet in cases and controls, which was significantly lower in ischemic stroke patients (*p* < 0.001). The number of cases with a high level of adherence was lower compared to controls (36 vs. 6). Regarding low adherence, 5 cases had low adherence compared to 0 controls. A total of 113 cases had low–moderate adherence compared to 38 controls, and 90 cases had high–moderate adherence compared to 140 controls.

Table 4 shows the level of adherence to the DASH diet and each of its factors in cases and controls. The total score for DASH diet adherence was significantly lower in cases (5.042 ± 1.486) compared to controls (6.654 ± 1.471), (*p* < 0.001). Considering each factor alone, adherence was significantly lower in cases compared to controls for all factors (*p* = 0.038 for sweets and *p* < 0.001 for all the remaining), except for meat, poultry, and fish, which did not show any significant difference (*p* = 0.46).

### 3.5. Stroke-Related Characteristics in Ischemic Stroke Patients

Table 5 shows the disability level, number of strokes and age at first diagnosis for the included cases. Around one-third of cases (65 cases; 30%) had moderate disability (mRS score = 3), around one-fourth (50 cases; 23.4%) had slight disability (mRS score = 2), and another one-fourth (46 cases; 21.5%) had moderate–severe disability (mRS score = 4). Only two cases (0.9%) had no symptoms and eight cases (3.7%) died. The majority of patients that were enrolled had only one stroke (151 participants, 70.6%), while one-fourth had two strokes (23.8%), and the average age at the first stroke was 67.724 ± 13.284.

### 3.6. Effect of DASH Diet Adherence on Disability Level (mRS) in Ischemic Stroke Patients

Table 6 shows the association between socio-demographic characteristics, pre-existing health-related factors, DASH diet adherence level, and disability level in ischemic stroke patients.

Higher ages were associated with a higher mRS score, corresponding to a higher disability level (*p* < 0.001). Similarly, the percentage of those with hypertension, hyperlipidemia, DVT or PE, atrial fibrillation, and migraine was higher in patients with high disability levels compared to those with lower disability levels (*p* = 0.023 for DVT or PE and *p* < 0.001 for the rest).

DASH diet adherence and the level of disability (mRS score) showed a significant association (*p* = 0.018). A total of 60% of those with low adherence to the DASH diet had an mRS score between 5 and 6, compared to 0% and 40% for those with scores of 3–4 and 0–2, respectively. Of those with high adherence to the diet, 66.67% had an mRS score of 0–2, 16.67% had an mRS score of 3–4, and 16.67% had an mRS score of 5–6.

### 3.7. Multivariate Analysis: Logistic Regression

Table 7 shows the bimonial regression regarding the incidence of ischemic stroke. Being divorced (*p* = 0.036, OR: 3.884, CI95% 1.092–13.818), being non-educated (*p* = 0.004, OR: 2.713, CI95% 1.38–5.333), having no education following school level (*p* < 0.001, OR: 5.602, CI95%2.369–13.246), being a smoker (*p* = 0.035, OR: 1.885, CI95% 1.044–3.402), having a family history of stroke (*p* < 0.001, OR: 4.707, CI95% 2.48–8.934), having hypertension (*p* < 0.001, OR: 6.536, CI95% 3.093–13.81), having atrial fibrillation (*p* < 0.001, OR: 5.828, CI95% 2.647–12.831), and lower adherence to the DASH diet (*p* < 0.001, OR: 0.526, CI95% 0.428–0.645) were all associated with a higher incidence of ischemic stroke.

Table 8 shows the multinomial regression regarding the disability level in stroke patients. Having atrial fibrillation versus not having atrial fibrillation (*p* < 0.001, OR: 10.286, CI95% 3.07–34.465) and low versus high DASH diet adherence (*p* = 0.041, OR: 44.263, CI95% 1.163–1684.246) were significantly associated with an increased likelihood of having moderate disability levels (mRS 3–4) compared to those with low disability levels (mRS 0–2).

Having no employment versus a free profession (non-employed workers) (*p* < 0.001, OR: 3.692, CI95% 1.051–12.977), having atrial fibrillation versus not having atrial fibrillation (*p* = 0.007, OR: 4.075, CI95% 1.468–11.307), having migraine versus not having migraine (*p* < 0.001, OR: 0.069, CI95% 0.019–0.252), and low versus high DASH diet adherence (*p* = 0.041, OR: 2.49 × 10^−8^, CI95% 0–2.49 × 10^−8^) were significantly associated with an increased likelihood of having high disability levels (mRS 5–6) compared to those with low disability levels (mRS 0–2).

## 4. Discussion

This case–control study assessed the effect of some health-related factors on ischemic stroke incidence and disability levels, focusing on the effect of DASH diet adherence. Smoking, family history of stroke, hypertension, hyperlipidemia, atrial fibrillation, and myocardial infarction were associated with a higher risk of stroke, and hypertension, hyperlipidemia, DVT/PE, atrial fibrillation, and migraine were associated with a higher disability level. Furthermore, the results of this case–control study demonstrated a protective role for the DASH diet regarding the incidence of ischemic stroke, as cases had lower adherence to the DASH diet compared to controls. In addition, higher adherence to the DASH diet was found to be associated with a lower level of disability.

Similar to this study, several studies previously reached similar results regarding the increased risk of ischemic stroke caused by smoking, family history of ischemic stroke, dyslipidemia, atrial fibrillation, and MI (*p* < 0.001 for all factors) [22]. Smoking has a dose–response relationship with ischemic stroke, where lower smoking levels are associated with lower stroke levels [23]. Smoking is believed to cause this risk by acting on other risk factors, such as inducing atherosclerosis and leading to hypercoagulable states [24,25]. A family history of ischemic stroke is also correlated with higher risk; several studies have confirmed the presence of a genetic component in ischemic stroke [26]. Hyperlipidemia is also considered as one of the major risk factors, as it leads to the formation of plaque inside the blood vessels, leading to an increased risk of MI or stroke [27]. MI was also found to be associated with a higher incidence of stroke; the similarity between risk factors such as smoking and dislipidemia leads to their co-occurrence [28]. Around 9% of ischemic stroke patients have a silent MI, and the highest risk of stroke occurs 5 days after a heart attack [29,30]. In addition to hyperlipidemia, atrial fibrillation is linked to both MI and stroke by causing blood clots, increasing the risk of stroke by 5-fold [31].

On the other hand, this study found no significant correlation between DVT/PE, migraine and stroke incidence (*p* = 0.111 and *p* = 0.21, respectively). These is some contradiction in the literature, where some studies have found a positive correlation, while others did not [32]. Several studies suggested that DVT/PE are complications commonly seen in ischemic stroke patients [33], while the risk of ischemic stroke occurring after a DVT or PE remains low, ranging between 1 and 10 percent [34]. As for migraine, despite its possible co-occurrence with ischemic stroke, there is no strong evidence considering migraine as a risk factor. When ischemic stroke occurs during migraine attacks, it is differentiated from other strokes and called Migraneous Infarction, with a low incidence of 0.8/100,000/year [35]. Further investigation is needed to better understand this relationship.

As for hypertension, this study confirms its correlation with a higher rate of ischemic stroke (90.65% of cases; *p* < 0.001) and higher disability levels (95.35% of those with severe disability; *p* < 0.018). The results also showed that better adherence to the DASH diet was associated with a lower rate of severe disability (mRS 5–6). Only 16.67% of those with high adherence to the DASH diet had severe disability, compared to 19.21% of those with moderate adherence, and 60% for those with low adherence (*p* = 0.018).

Previous studies classified hypertension as the number one risk factor for stroke, being present in around 84% of cases [4]. Therefore, preventing and controlling hypertension are the main approaches to prevent stroke and minimize any resulting disability [36]. Of the multiple ways to control blood pressure, diet remains one of the most important factors, acting acutely and in the long-term [37]. Thus, those susceptible to ischemic stroke could benefit from antihypertensive diets.

The DASH diet consists of consuming vegetables, fruits, carbohydrates, low-fat dairy products, lean meat products, nuts, and grains, with a significant reduction in sodium intake [7]. It is designed to lower high blood pressure by decreasing the amount of saturated fat and salt intake [9]. It also has a natriuretic effect by interacting with the renin–angiotensin–aldosterone system (RAAS). This interaction has a hypotensive effect by stimulating hormonal and vascular responses [38]. Based on these effects, the DASH diet could also be of value in pharmacological interventions through managing hypertension [37].

Several mechanisms are believed to interact, leading to the prevention of ischemic stroke, following a high level of adherence to the DASH diet. The decrease in blood pressure is considered the main reason for the reduction in stroke incidence, but the DASH diet was also found to reduce lipid levels and body weight, which also contribute to this reduction [39,40]. Furthermore, metabolic syndrome and diabetes mellitus 2, which are linked to the pathology of ischemic stroke, were decreased following high adherence to the DASH diet [41,42,43]. Moreover, the DASH diet was found to decrease inflammation and to have an antioxidant role, both of which are believed to contribute to the pathophysiology of ischemic stroke [44,45].

Other studies assessed the effect of the DASH diet on stroke incidence. For instance, a study on a Chinese cohort found that adherence to the DASH diet was beneficial for reducing BP and stroke rates in the long term [46]. Similarly, a case–control study in Iran found that the rate of stroke was lower in those with higher levels of DASH diet adherence [47]. The results of these studies align with our findings, which favor a protective role for the DASH diet, with no studies to this day, to our knowledge, disagreeing with the above-mentioned role of DASH.

Compared to other diets, an advantage of the DASH diet is its conventional guidelines on the size and number of servings [48]. The DASH diet led to a significantly lower BP compared to those following a regular diet with restricted sodium [49]. In addition to its function in lowering blood pressure, it appeared to play a role in preventing the development of hypertension [9]. Several studies demonstrated that better blood pressure was recorded after following the DASH diet compared to a regular diet in patients at risk of hypertension [7]. The modification of the DASH diet’s macronutrients by increasing proteins and removing saturated fat had a favorable effect on maintaining an optimal BP and lowering systolic and diastolic blood pressure [50]. Furthermore, the 10-year Framingham score used for assessing cardiovascular risks was also reduced by approximately 13% in patients following a DASH diet [51]. Consequently, the 2013 AHA/ACC Guidelines considered the DASH diet as a dietary pattern for reducing BP and LDL-C [52]. 

A commitment to a healthy lifestyle based on a healthy diet, such as the DASH diet, reduces the risk of stroke in patients at risk and modifies the impact of comorbidity by ameliorating the disability score in stroke patients [47,53]. These findings support our results, which showed milder disability in stroke patients with a high adherence to the DASH diet. Although the DASH diet meets the global recommendations and demonstrated promising results in adherent patients, there are no clear studies on its long-term effectiveness and further studies should focus on the dietary adherence rate, in addition to providing a clear pathway to explaining how the DASH diet exerts its effect.

This study has some limitations that are worth mentioning. Due to the case–control nature of this study, the potential for recall bias is present, as participants may have recalled their dietary habits with a lack of precision. The cognitive impairment that might result from stroke may also affect the recall process. In addition, this study lacked a timing assessment when considering DASH diet adherence. Moreover, the matching between cases and controls was based on age and gender, disregarding other factors. For instance, educational level was significantly different between cases and controls, which might affect the credibility of the self-reported information. Furthermore, the majority of the included sample had moderate adherence to the DASH diet, with only a few having high or low adherence. This could limit the understanding of the effect of the DASH diet on disability rates. Further studies with other study designs are required to overcome such limitations, such as prospective cohort studies.

## 5. Conclusions

This study demonstrated a protective role for the DASH diet regarding ischemic stroke, and links adherence to this diet to a lower level of disability. Furthermore, this study confirmed that smoking, family history of ischemic stroke, hypertension, hyperlipidemia, atrial fibrillation, and myocardial increase the risk of stroke, and hypertension, hyperlipidemia, DVT/PE, atrial fibrillation, and migraine lead to a higher disability level. This role highlights the necessity for strict adherence to the DASH diet, especially in patients with higher susceptibility to ischemic stroke. Further studies are needed to better understand the mechanistic role of the DASH diet in preventing ischemic stroke, and to reach evidence-based guidelines regarding dietary approaches in patients with specific risk factors for ischemic stroke. The correlation between DASH diet adherence and specific treatments could also be of great benefit in managing ischemic stroke cases.

## Figures and Tables

**Table 1:** Bivariate analysis of socio-demographic characteristics associated with ischemic stroke.

Factor	Category	Cases		Control		*p* Value
		Number	Percentage	Number	Percentage	
Gender	Female	103	48.13	106	49.53	0.772
	Male	111	51.86	108	50.47	
Marital Status	Single	13	6.07	20	9.34	<0.001 *
	Married	119	55.60	149	69.63	
	Divorced	5	2.34	4	1.87	
	Widowed	77	35.98	41	19.16	
Education	Not educated	51	23.83	83	38.60	<0.001 *
	School education	99	46.26	78	36.45	
	Non-healthcare education	60	28.04	36	16.82	
	Healthcare education	4	1.87	17	7.94	
Employment	Not employed	152	71.03	147	68.69	0.65
	Employed	31	14.48	29	13.55	
	Free profession	31	14.48	38	17.76	
Age	Mean + SD	68.589 ± 13.436		66.841 ± 14.488		0.196

* represents *p* < 0.05.

**Table 2:** Bivariate analysis of socio-demographic characteristics associated with DASH diet adherence score.

Factor	Category	DASH Score	*p* Value
		Mean ± SD	
Gender	Female	6.16 ± 1.611	<0.001 *
	Male	5.55 ± 1.7	
Marital Status	Single	5.879 ± 1.653	0.976
	Married	5.823 ± 1.722	
	Divorced	5.778 ± 1.563	
	Widowed	5.903 ± 1.629	
Education	Not educated	6.071 ± 1.471	0.044 *
	School education	5.805 ± 1.631	
	Non-healthcare education	5.51 ± 1.998	
	Healthcare education	6.333 ± 1.599	
Employment	Not employed	5.992 ± 1.652	0.026 *
	Employed	5.475 ± 1.812	
	Free profession	5.551 ± 1.638	
Age	R (R squared)	0.084 (0.007)	0.084

* represents *p* < 0.05.

**Table 3:** Bivariate analysis of pre-existing health-related conditions on ischemic stroke.

Factor	Category	Cases		Control		*p* Value
		Number	Percentage	Number	Percentage	
Smoking	Yes	126	58.88	88	41.12	<0.001 *
	No	88	41.12	126	58.88	
Family history of stroke	Yes	105	49.06	29	13.55	<0.001 *
	No	109	50.94	185	86.45	
Hypertension	Yes	194	90.65	107	50	<0.001 *
	No	20	9.35	107	50	
Hyperlipidemia	Yes	151	70.56	81	37.85	<0.001 *
	No	63	29.44	133	62.15	
DVT or PE	Yes	27	11.21	17	7.94	0.111
	No	187	87.39	197	92.06	
Atrial fibrillation	Yes	74	34.58	13	6.07	<0.001 *
	No	140	65.42	201	93.93	
Migraine	Yes	28	13.08	27	12.62	0.21
	No	186	86.92	187	87.38	
Myocardial infarction	Yes	49	22.90	17	7.94	<0.001 *
	No	165	77.10	197	92.06	

* represents *p* < 0.05.

**Table 4:** Bivariate analysis of DASH diet adherence associated with ischemic stroke.

Factor	Category	Cases		Control		*p* Value
		Number	Percentage	Number	Percentage	
Total grain	>7	21	9.81	41	19.16	<0.001 *
	2–3	128	59.81	59	27.57	
	<5	65	30.38	114	53.27	
Whole grain	>2	39	18.22	56	21.17	<0.001 *
	1	64	29.90	98	45.79	
	<1	111	5.14	60	28.04	
Vegetables	>4	57	26.63	116	54.21	<0.001 *
	2–3	135	63.09	73	34.11	
	<2	22	10.28	25	11.68	
Fruits	>4	41	19.16	101	47.20	<0.001 *
	2–3	100	46.73	76	35.51	
	<2	73	34.11	37	17.29	
Dairy foods	>2	82	38.32	121	56.54	<0.001 *
	1	104	48.60	63	24.44	
	<1	28	13.08	30	14.02	
Meat, poultry, and fish	>2	61	28.50	69	32.24	0.46
	3	100	46.73	102	47.66	
	>4	53	24.77	43	20.09	
Nuts, seeds, and dry beans	>4	60	28.04	41	19.16	<0.001 *
	2–3	98	45.79	76	35.51	
	<2	56	26.17	97	45.33	
%Kcal from fat	<30%	80	37.38	26	12.15	<0.001 *
	31–32	110	51.41	81	37.85	
	>33	24	11.21	107	50	
%Kcal from saturated fatty acids	<10	84	39.25	24	11.22	<0.001 *
	11–12	104	48.60	76	35.52	
	>13	26	12.15	114	53.27	
Sweets	<5	36	16.82	36	16.82	0.038 *
	6–7	74	34.58	51	23.83	
	>8	104	48.60	127	59.35	
Sodium	>1500	113	52.80	29	13.55	<0.001 *
	1501–2400	91	42.52	63	29.44	
	>2400	10	4.68	122	57.01	
Total Score	Mean + SD	5.042 ± 1.486		6.654 ± 1.471		<0.001 *

* represents *p* < 0.05.

**Table 5:** Descriptive analysis of stroke-related characteristics in ischemic stroke patients.

Factor	Category	Number	Percentage
mRS score	0	2	0.9
	1	8	3.7
	2	50	23.4
	3	65	30.4
	4	46	21.5
	5	35	16.4
	6	8	3.7
Number of strokes	1	151	70.6
	2	51	23.8
	3	10	4.7
	4	2	0.9
Age	Mean ± SD	68.589 ± 13.436	
Age at first stroke	Mean + SD	67.724 ± 13.284	

**Table 6:** Bivariate analysis of socio-demographic and health-related factors, and DASH diet adherence, and their association with disability level (mRS) in ischemic stroke.

Factor	Category	mRS Category			Total	*p* Value
		0 until 2	3 until 4	5 until 6		
Gender	Female	27 (26.21%)	54 (52.43%)	22 (21.36%)	103	0.816
	Male	33 (29.73%)	57 (51.35%)	21 (18.92%)	111	
Marital Status	Single	2 (15.38%)	8 (61.54%)	3 (23.08%)	13	0.003 *
	Married	38 (31.93%)	66 (55.46%)	15 (12.61%)	119	
	Divorced	4 (80%)	1 (20%)	0 (0%)	5	
	Widowed	16 (20.78%)	36 (46.75%)	25 (32.47%)	77	
Education	Not educated	6 (11.76%)	27 (52.94%)	18 (35.3%)	51	0.001 *
	School education	25 (25.25%)	57 (57.57%)	17 (17.18%)	99	
	Non-healthcare education	27 (45%)	26 (43.33%)	7 (11.67%)	60	
	Healthcare education	2 (50%)	1 (25%)	1 (25%)	4	
Employment	Not employed	32 (21.05%)	84 (55.26%)	36 (23.69%)	152	0.004 *
	Employed	16 (51.61%)	13 (41.94%)	2 (6.45%)	31	
	Free profession	12 (38.71%)	14 (45.16%)	5 (16.13%)	31	
Age	Mean + SD	63.15 + 15.741	69.36 + 11.952	74.186 + 10.839	68.589 + 13.436	<0.001 *
Smoking	Yes	37 (29.37%)	71 (56.35%)	18 (14.28%)	126	0.038 *
	No	23 (26.14%)	40 (45.45%)	25 (28.41%)	88	
Family history of stroke	Yes	31 (29.52%)	53 (50.48%)	21 (20%)	105	0.887
	No	29 (26.61%)	58 (53.21%)	22 (20.18%)	109	
Hypertension	Yes	49 (25.25%)	104 (53.61%)	41 (21.14%)	194	0.018 *
	No	11 (55%)	7 (35%)	2 (10%)	20	
Hyperlipidemia	Yes	31 (20.53%)	87 (57.61%)	33 (21.85%)	151	0.001 *
	No	29 (46.03%)	24 (38.1%)	10 (15.87%)	63	
DVT or PE	Yes	3 (11.11%)	14 (51.85%)	10 (37.04%)	27	0.023 *
	No	57 (30.48%)	97 (51.87%)	33 (17.65%)	187	
Atrial fibrillation	Yes	10 (13.51%)	41 (55.41%)	23 (31.08%)	74	<0.001 *
	No	50 (35.71%)	70 (50%)	20 (14.29%)	140	
Migraine	Yes	20 (71.43%)	8 (28.57%)	0	28	<0.001 *
	No	40 (21.51%)	103 (55.38%)	43 (23.11%)	186	
Myocardial infarction	Yes	10 (20.41%)	25 (51.02%)	14 (28.57%)	49	0.165
	No	50 (30.3%)	86 (52.12%)	29 (17.58%)	165	
DASH adherence	Low	2 (40%)	0 (0%)	3 (60%)	5	0.018 *
	Moderate	54 (26.60%)	110 (54.19%)	39 (19.21%)	203	
	High	4 (66.68%)	1 (16.67%)	1 (16.67%)	6	

* represents *p* < 0.05.

**Table 7:** Multivariable analysis: Binomial regression regarding the incidence of ischemic stroke.

Independent Variables	*p* Value	OR	CI 95%	
			Lower Bound	Upper Bound
Marital Status	0.03 *			
Single	0.488	1.540	0.455	5.212
Married	0.293	3.087	0.378	25.212
Divorced	0.036 *	3.884	1.092	13.818
Educational Level	0.01 *			
Not educated	0.004 *	2.713	1.38	5.333
School education	<0.001 *	5.602	2.369	13.246
Non-healthcare-related education	0.788	1.228	0.274	5.497
Smoking	0.035 *	1.885	1.044	3.402
Family History of Stroke	<0.001 *	4.707	2.48	8.934
Hypertension	<0.001 *	6.536	3.093	13.81
Hyperlipidemia	0.285	1.397	0.757	2.578
DVT or PE	0.692	0.822	0.311	2.17
Atrial fibrillation	<0.001 *	5.828	2.647	12.831
Myocardial Infarction	0.444	1.37	0.611	3.073
DASH score	<0.001 *	0.526	0.428	0.645
Constant	0.659	0.656		

Dependent variable: cases vs. controls; * represents *p* < 0.05.

**Table 8:** Multivariable analysis: Multinomial regression of the disability level in ischemic stroke patients.

Independent Variables	*p* Value	OR	CI 95%	
			Lower Bound	Upper Bound
Model 1: mRS 3–4 vs. mRS 0–2				
Age	0.154	0.950	0.886	1.019
Single vs. widowed	0.586	2.224	0.126	39.344
Married vs. widowed	0.300	0.454	0.102	2.022
Not educated vs. HC-related education	0.920	0.812	0.014	47.219
School education vs. HC-related education	0.391	0.182	0.004	8.879
Non-HC vs. HC education	0.122	0.049	0.001	2.244
Not employed vs. free profession	0.784	1.273	0.226	7.169
Employed vs. free profession	0.369	0.353	0.036	3.419
Smoker	0.747	0.821	0.248	2.723
Hypertensive	0.609	1.779	0.195	16.185
Hyperlipidemic	0.209	2.227	0.639	7.757
History of DVT or PE	0.401	2.072	0.367	11.707
History of atrial fibrillation	<0.001 *	10.286	3.07	34.465
History of myocardial Infarction	0.314	1.875	0.551	6.373
Low vs. high DASH adherence	0.041 *	44.263	1.163	1684.246
Moderate vs. high DASH adherence	0.246	5.431	0.312	94.642
Model 2: mRS 5–6 vs. mRS 0–2				
Age	0.115	0.96	0.912	1.01
Single vs. widowed	0.273	3.929	0.339	45.482
Married vs. widowed	0.440	1.579	0.496	5.03
Not educated vs. HC education	0.419	3.935	0.142	108.91
School vs. HC-related education	0.76	1.644	0.067	40.128
Non-HC vs. HC-related education	0.807	0.679	0.03	15.16
Not employed vs. Free profession	0.042 *	3.692	1.051	12.977
Employed vs. Free profession	0.624	1.381	0.38	5.024
Smoker	0.571	1.304	0.521	3.259
Hypertensive	0.737	1.259	0.327	4.843
Hyperlipidemic	0.055	2.513	0.981	6.44
History of DVT or PE	0.620	1.493	0.307	7.265
History of atrial fibrillation	0.007 *	4.075	1.468	11.307
History of migraine	<0.001 *	0.069	0.019	0.252
History of myocardial Infarction	0.842	0.899	0.315	2.561
Low vs. high DASH adherence	0.041 *	2.49 × 10^−8^	0	2.49 × 10^−8^
Moderate vs. high DASH adherence	0.059	12.668	0.91	176.427

Dependent variable: mRS categories; reference category: 0–2; * represents *p* < 0.05.

## Data Availability

Data are available upon request from corresponding author due to privacy.
